# Lessons Learned During the COVID-19 Pandemic in Central India: Overlooked Psychological and Social Determinants of Health

**DOI:** 10.7759/cureus.29686

**Published:** 2022-09-28

**Authors:** Nisha Kaithwas, Anant T Pawar, Rashmi Yadav, Sanjay H Ingle, Shruti G Sethia, Soumitra Sethia

**Affiliations:** 1 Psychiatry, Nandkumar Singh Chouhan Government Medical College, Khandwa, IND; 2 Community Medicine, Nandkumar Singh Chouhan Government Medical College, Khandwa, IND; 3 Psychiatry, Government District Hospital, Khandwa, IND; 4 Paediatrics, Nandkumar Singh Chouhan Government Medical College, Khandwa, IND

**Keywords:** covid 19 impact of lockdown, pandemic prevention and control, social issues, psychological issues, public heath, covid-19 retro

## Abstract

Background: The coronavirus disease 2019 (COVID-19) pandemic, which started in 2019, has created unprecedented public health problems, mental health crises, and economic and social problems. These effects have been studied by numerous researchers on the general population but none on hospitalized and discharged COVID-19 patients.

Aim: To assess psychological and social problems among hospitalized and discharged COVID-19 patients.

Methods: During lockdown and post-lockdown in India, we interviewed 500 COVID-19 patients admitted at our tertiary care center during their hospitalization and post-discharge period for psychological and social problems.

Results: The common psychological issues in hospitalized patients during lockdown were anxiety and misconceptions about COVID-19, while insomnia, anxiety, and frustration were common during the post-lockdown period. The typical social problems in hospitalized patients during the lockdown were containment-related issues, discrimination, longer wait for repeat COVID-19 tests, and boredom; whereas issues related to employment and financial matters were common during post-lockdown. Psychological problems comparatively decreased whereas social problems increased after discharge.

Conclusion: Unrehearsed mitigation strategies at the beginning of the pandemic unknowingly led to various psychological and social problems. It was further aggravated by a lack of information and miscommunication.

## Introduction

The coronavirus disease 2019 (COVID-19) pandemic is a global health and economic crisis. COVID-19 started in Wuhan, China, in early December 2019, came into the news in late December to early January 2020, and later was declared as an emergency in the third week of January 2020. The World Health Organization (WHO) declared this a Public Health Emergency of International Concern (PHEIC) on January 31, 2020, and was finally declared a pandemic on March 11, 2020 [1]. By the end of March 2020, more than 190 countries had been affected, with an exponential rise in the number of cases and deaths worldwide.

As per the WHO report 2020, only 46% of countries and territories had COVID-19 preparedness and response plan by March 1, 2020 [2]. Consequently, a wide fragment of the world’s unprepared population faced a myriad of unanticipated circumstances and issues, owing to nationwide lockdowns, and home-confinement strategies in order to prevent disease transmission. However, with the progression of the pandemic, with more people getting infected, hospitalized, losing loved ones, or facing social issues such as job loss, financial problems, stigma, and discrimination; it only seemed to be an unending crisis.

In India, a nationwide lockdown was implemented on March 25, 2020, following which the initial response was “coronaphobia” and “mass hysteria” comprising extreme panic, desperate hoarding, a social boycott of COVID-19 patients, and discriminatory behavior towards healthcare and other frontline workers [3]. With the gradual progression of the pandemic, it was observed that psychological and social effects were not merely due to the pandemic itself but also the prolonged lockdown and other similar circumstances like halted economic activities, unemployment, and loss of livelihood [4].

Similarly, many unforeseen social issues like quarantine, social isolation, and uncertainty begat various psychological problems like loneliness, impaired self-care, maladaptive coping strategies, and difficulty handling the parallel "infodemic" [5]. Ventriglio and Bellomo have also mentioned various reactions such as confusion, anger, defiant behavior, fear, suspiciousness, and discrimination among citizens [6]. The unpredictable nature and course of the pandemic caused fear of the unknown, leading to acute/chronic stress, panic, and many mental health problems. Soon after the pandemic, India also faced a significant increase in psychiatric problems, including a sudden rise in suicide [7].

There are many studies and online surveys conducted in India and worldwide, mainly studying the general population and healthcare workers for psychological and social issues during the pandemic; however, no epidemiological data on psychological and social problems of hospitalized and discharged COVID-19 patients are available from India. The management of any pandemic is insufficient and incomplete if psychological and social components, very crucial yet often overlooked health determinants, are not given the necessary attention.

## Materials and methods

This retrospective analysis was conducted to assess psychological and social problems among 500 hospitalized and discharged COVID-19 patients of Nandkumar Singh Chouhan Government Medical College (NSCGMC), Khandwa, Madhya Pradesh, India. The study was done over a period of six months, from April 2020 to September 2020. Institutional ethics committee permission was obtained (letter no. 16/IEC/NSCGMCK/2022 dated January 24, 2022).

In India, the initial strategy to tackle the pandemic was to establish three-tier COVID-19 care facilities namely: COVID-19 care centres (CCC) for mild cases, dedicated COVID-19 health centres (DCHC) for moderate cases, and dedicated COVID-19 hospitals (DCH) for severe cases. Our facility had all three types. Since the outset of the pandemic, many admitted COIVD-19 patients were having various psychological issues. A few incidences of non-cooperation, aggression, absconding from COIVD-19 wards and threatening behaviour were also reported. To minimize psychological burden and to provide psychological support, teleconsultation service was started by the Psychiatry department of NSCGMC from April 2020 till September 2020, as per instructions by the administration. The detailed records of psychological and social problems of admitted and discharged patients and their management were entered in Microsoft Excel spreadsheets (Microsoft Corporation, Redmond, Washington, United States). These records were maintained for reporting purposes and for communicating with other established response teams for effective and holistic pandemic management at our place.

During the six-month period, a total of 516 patients were consulted and counselled during their hospitalization and till 15 days after discharge. It was a convenience sampling of 500 patients (61 from DCH and 439 from CCC and DCHC); as the records of 16 patients were either incomplete or couldn’t be followed up, hence they were excluded. Demographic details of admitted COIVD-19 patients, such as name, age, sex, address, date of admission, contact number of patient and their relatives were collected at the time of admission only, by the record-keeping department of NSCGMC. Additionally, other clinical information like past psychiatric history (substance use, psychiatric treatment etc.) and date of discharge were collected from patients and their relatives. These patients were consulted daily by psychiatrists/authors via audio or video calling. A few cases were consulted in person, such as those who were reluctant to talk on the phone, agitated and psychotic patients, or when the treating doctor requested for in-person psychiatric consultation. During the hospital stay and post-discharge period, each patient was interviewed at least once a day by either psychiatrist, comprising more than 10,000 interviews during the study period of six months.

The assessment for the psychological and social problems was done by open-ended interviews, without using any specific structured questionnaire or scale. Corroborative details whenever needed, for assessment of behavioural or psychological problems, were collected through on-duty, treating, or ward in-charge doctors and nursing staff. Psychiatry diagnosis was made clinically (history, self-report, observation in the ward, and mental status examination) as per the International Classification of Diseases, 10th revision (ICD-10) and Diagnostic and Statistical Manual of Mental Disorders, fifth edition (DSM-5),. Whenever needed, supportive counselling was done and if necessitated, psychiatric treatment was provided. These patients were interviewed by two psychiatrists; hence, the chances of subjective bias in diagnosis, transference, and countertransference were minimal.

The recorded data were entered in a Microsoft Excel spreadsheet and analyzed using descriptive statistics. The data were presented as frequencies and percentages.

## Results

In our study, we retrieved and analyzed the data of 500 COVID-19 patients. The mean age of the study participants was 38.29 ± 17.5 years; most patients belonged to the age group of 21-30 years. A total of 121 participants were above 50 years of age and 79 were below 20 years of age. A total of 303 participants were male and 80.4% were living in urban areas (Table 1).

**Table 1:** Distribution of participants according to sex and age groups

Age Group	Female	Male	Total
Up to 10 Years	7 (3.5%)	10 (3.3%)	17 (3.4 %)
11 to 20 Years	29 (14.7%)	33 (10.9%)	62 (12.3%)
21 to 30 Years	49 (24.8%)	74 (24.4%)	123 (24 %)
31 to 40 Years	32 (16.2%)	62 (20.4%)	94 (18.6%)
41 to 50 Years	31 (15.7%)	52 (17.2%)	83 (16.4%)
51 to 60 Years	26 (13.2%)	38 (12.5%)	64 (12.6%)
61 to 70 Years	16 (8.1%)	23 (7.6%)	39 (7.7%)
71 to 80 Years	6 (3.0%)	10 (3.3%)	16 (3.2%)
81 Years and above	1 (0.5%)	1 (0.3%)	2 (0.4%)
Total	197 (100%)	303 (100%)	500 (100 %)

Problems during hospitalization

Psychological Problems

Anxiety (27%) was the most common psychological problem, followed by insomnia (12.8%) and depression (3.8%). Substance use-related issues were found in 2.4% of the patients, somatic symptoms in 3%, and grief in 1.4% of patients. The majority of patients didn't fulfil all the diagnostic criteria for a psychiatric disorder according to guidelines (ICD-10 and DSM-5). However, common themes or patterns of concern were found such as 11.4% of patients showed behavioural issues like anger, frustration, irritation, and non-cooperation, 10.8% had misconceptions about COVID-19 infection, and 9% were excessively worried about their family members isolated at home. Of all, 37.6% needed supportive counselling and 23.8 % needed treatment, either short or long-term (Table 2).

**Table 2:** Psychological problems and concerns faced by COVID-19 patients during hospitalization and after discharge COVID-19: coronavirus disease 2019

Psychological problems	During hospitalization	After discharge
Anxiety	135 (27%)	100 (20%)
Insomnia	64 (12.8%)	28 (5.6%)
Depression	19 (3.8%)	-
Substance use disorder (e.g. withdrawal)	12 (2.4%)	-
Somatic symptoms	15 (3%)	85 (17%)
Grief	7 (1.4%)	6 (1.2%)
Behavioural and emotional problems (anger/frustration)	57 (11.4%)	73 (14.6%)
Misconception about COVID-19	54 (10.8%)	-
Worry for family members	45 (9%)	-

Social Problems

The most common social issue during hospitalization was “high expressed emotion” (17%) of family members like calling repeatedly for updates on the patient’s health and needing reassurance repeatedly. Various containment-zone-related issues like non-access to daily necessities by their family were reported by 13.6% of hospitalized patients, 11% of patients reported significant distress due to a longer wait for the report of repeat COVID-19 test, 10% of the patients, mainly females, reported concern that nobody else is there at home to do house chores. Stigma and discrimination (like verbal comments and taunts, not allowing their family members to use common toilets, water sources, or even residential /colony gates) were reported by 6.4% of patients. Among the various social issues, 7% of patients showed mistrust and misbehaviour towards medical staff such as being non-compliant with the treatment, spreading rumours or false videos, being verbally abusive, or showing physical aggression. Disobeying COVID-19 protocols like mask wearing and social distancing was found in 3.4% of patients. Delayed result and a longer waiting period for the report of COVID-19 test of close contacts and family members was reported by 6.2 % of patients (Table 3).

**Table 3:** Social problems and concerns faced by COVID-19 patients during hospitalization and after discharge COVID-19: coronavirus disease 2019

Social problems	During hospitalization	After discharge
High expressed emotions of relatives	85 (17%)	-
Containment (no access to milk, vegetables etc.)	68 (13.6%)	150 (30%)
Longer wait for repeat COVID-19 test report	55 (11%)	-
Nobody to do house chores, like cooking/ taking care of children	50 (10%)	12 (2.4%)
Discriminatory behavior and stigma	32 (6.4%)	29 (5.8%)
Misbehave and mistrust towards medical staff	33 (7%)	-
Not following social distancing, wearing mask, or cooperating with treatment	17 (3.4%)	-
Late COVID-19 test/result of family members	31 (6.2%)	-
Boredom/feeling of loneliness due to social restriction	-	90 (18%)
Financial issue, job loss, loss in business	-	87 (17.4%)
COVID-19 certificate needed for job leave/fitness/ exam	-	28 (5.6%)
Difficulty in social isolation due to less number of rooms at home	-	22 (4.4%)

**Figure 1:**
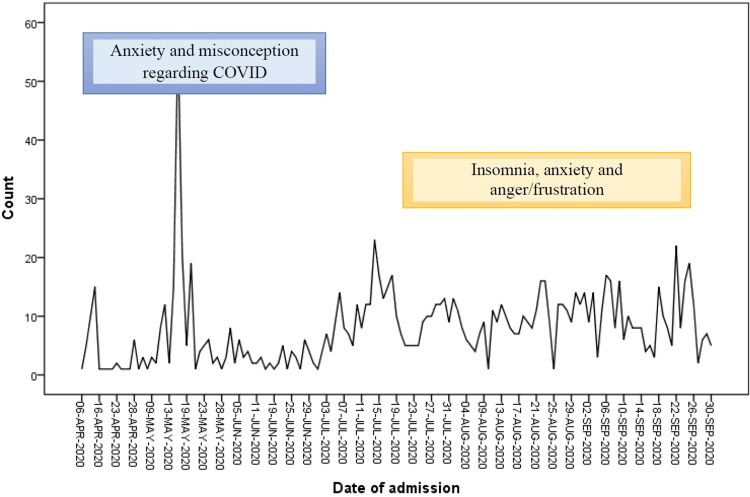
Most common psychological problems faced by hospitalized patients during lockdown and post lockdown (Counts- Number of Patients)

The most common psychological problems in hospitalized patients, during April-June 2020 (lockdown), were anxiety and misconception about COVID-19. While problems faced during July-September 2020 (post lockdown) were insomnia, anxiety, and frustration (Figure 1).

**Figure 2:**
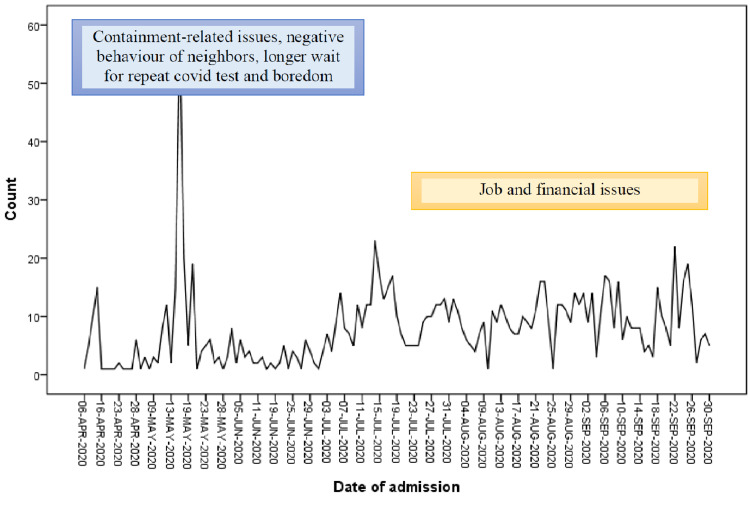
Most common social problems faced by hospitalized patients during lockdown and post lockdown (Count- Number of Patients)

The social problems in hospitalized patients during lockdown were containment-related issues; stigma and discrimination, longer wait for repeat COVID-19 tests and boredom, whereas during post-lockdown, they were job and financial-related matters (Figure 2).

Problems after discharge

Psychological Problems

The most common psychological problem after discharge was anxiety (20%), of which 10.8% was related to job/financial matters. Somatic symptoms, excessive concern for minor bodily symptoms, and worry for recurrence were found in 17% of patients. Behavioural and emotional issues of frustration, feeling of rejection, loneliness, being locked up at home, and social restrictions were found in 14.6%. Insomnia was found in 5.6% of patients.

Social Problems

The most common social problem after discharge was containment-related issues (30%), of which 4.6 % were related to the inability to get daily necessities such as milk and vegetables and 25.4% due to containment lasting longer than expected. Issues related to social restrictions were reported by 18% of patients. Financial matter-related issues were reported by 17.4% of patients and stigma and discrimination by 5.8% of patients. A few patients (5.6%) needed the medical certificate for various purposes such as exemption or to appear in exams, and to join the workplace. Difficulty in social isolation due to few rooms at home was reported by 4.4% of patients and domestic issues like difficulty getting help for household chores were reported by 2.4% of patients.

The most common psychological issues after discharge, during lockdown and post lockdown, were similar; these were somatic symptoms and emotional reactions to social restriction. The social issues faced during lockdown were financial problems, containment-related issues, stigma, and discrimination.

## Discussion

With the outbreak of COVID-19 pandemic, almost all the countries had adopted various strategies such as lockdown, travel restrictions and containment to curtail the spread of infection. India also imposed a lockdown quite early (March 25, 2020), within two weeks of the declaration of COVID‑19 as a pandemic. Although at the inception, these strategies were presumed to be the best possible options against the rampant rise in COVID-19 cases. However, little was known about the additional burden these were going to create on the already existing COVID-19 burden. They had a widespread impact on the psyche and daily living of the people, especially those who were suspected or diagnosed with COVID-19 infection and were kept in isolation. In this regard, our study was planned to evaluate the psychological and social problems faced by patients infected with COVID-19, who are presumed to be most likely affected.

We searched various studies assessing psychological and social problems among COVID-19 patients but very few were available. Most of the researchers from India conducted online surveys on the general population [8-15], or among healthcare workers [16-17]. In a study by Joshi A, mentioning iCALL, a national-level technology-assisted psycho-social counselling service and field action project of the Tata Institute of Social Sciences, India, the author described various psychosocial issues of 349 genuine calls received from April 18, 2020, to May 18, 2020 [18]. To the best of our knowledge, There is no study on COVID-19 patients from India although we could find four studies on COVID-19 patients from China [19-22].

In our study, as presumed, elderly patients commonly presented with serious conditions and needed intensive care. Hence, most were not available for routine psychiatric interviews. Psychiatric consultation of such patients was done only on as and when needed basis. Consequently, most of our participants were in their third decade of age. Another possible argument can also be that India has a mostly young population (65% aged <35 years) [23].

During hospitalization, 422 patients (84.4%) had psychological problems and 347 (69.4%) reported social problems. Grover et al. conducted an online survey of the psychological impact of COVID-19 among the general population in India during the lockdown and found that about 70% of participants had poor mental well‑being [8]. Panigrahi et al. also did an online survey of 1537 participants from India and reported psychological distress in 47% [11]. Our sample was hospitalized patients, hence, higher the prevalence. After discharge, 288 patients (57.6%) had psychological problems and 390 (78%) reported social problems, possibly due to a decrease in hospitalization-related stressors and confrontation with the various social issues such as containment, lockdown, financial stress, etc. occurring after the discharge.

The most common psychiatric issue during hospitalization was anxiety (27%). Of the patients with anxiety, 16.4% were having reactive anxiety, which could be explained as a psychological response to unexpected, unpredictable stressors i.e. COVID-19 infection and the remaining 10.6% of the anxiety patients were worried well, which reflects high illness anxiety and maladaptive coping mechanisms in the early phase of the pandemic. Grover et al. also found moderate anxiety in 23.7% of the general population [8]. Similarly, Nagabhirava et al. found anxiety in 21.5% [12] and Verma et al. found anxiety in 28% of participants from India [9]. An online survey from China by Amjed et al. evaluating the psychological problems among the Chinese people (n=1074) during COVID-19 epidemic peak and subsequent lockdown, reported anxiety in 29% of participants [24].

Apart from this, 9% of our admitted patients, mostly middle-aged, were worried for their family members. The main concerns were fear of the spread of infection to others, various COVID-19 and non-COVID-19 related health needs, non-access to daily necessities, and financial troubles, especially among daily wagers. Anxiety disorders decreased from 27% during admission to 20% after discharge, possibly due to the change in stressful environment in the hospital to a more emotionally supportive and caring environment at home. Of these discharged patients, 10.8% were anxious mainly due to financial matters, which was expected owing to lockdown, and halting of major economic activities. Joshi found similar concerns among a sample of 348 callers [18]. The author reported health concerns in 6.3%, livelihood and financial concerns in 8.9%, and practical concerns (food, transportation, essential services etc.) in 30.7%.

Insomnia was found in 12.8% of admitted patients, mostly the elderly, where pre-existing age-related sleep problem was aggravated by the sudden stressors of COVID-19 diagnosis, confinement in a hospital environment, and digital illiteracy resulting in an inability to connect with relatives. Roy et al. also found insomnia in 12% of the participants [10]. Most of our patients improved after short-term treatment or supportive counselling. Insomnia decreased from 12% during hospitalization to 5.6% after discharge, possibly due to similar reasons as that for anxiety. Grover et al. found markedly decreased sleep in 6.2%; however, their sample was from the general population contrary to ours [8].

During hospitalization, 11.4% of patients showed behavioural issues like anger, frustration and non-cooperation, 10.8% had misconceptions about covid-19 infection, and 9% were excessively worried about their family members isolated at home. Joshi also described similar concerns, themes and subthemes in their study [18].

We found depression in 3.8% of hospitalized patients; mostly reported stressors like a recent death in the family or multiple family members infected with COVID-19. Depression was common in patients with chronic co-morbidities. These are proven predisposing or precipitating risk factors for depression. Grover et al. also found moderate to severe depression in 3% population [8]. Similarly, patients in grief (1.4%), with a recent loss of a family member, had a predominant psychological theme of blaming self or the government for inadequate health facilities and bargaining.

Somatic symptoms in hospitalized patients (3%) increased significantly to 17% after discharge. This could be attributed to the novel nature of COVID-19 infection, frequently-changing government guidelines, and difficulty in access to medical services at home, leading to preoccupation and frequent exaggeration of minor bodily symptoms and hypervigilant behaviour [25].

There wasn’t much difference in behavioural issues during hospitalization (11.4%) and after discharge (14.6%), as behavioural issues were mostly found among young or middle-aged patients, due to the reasons of social restrictions and financial problems which didn't change much even after discharge. As Ventriglio and Bellomo have stated, restrictions and changing norms can lead to confusion, anger, and defiant behaviours [6].

The common psychological issues noted during lockdown were anxiety, insomnia and misconceptions about COVID-19 infection, which was quite expected due to the fear of the unknown and “coronaphobia”, superadded by the rumours, misinformation, etc. Shigemura et al. mentioned that overwhelming and sensational news headlines and images and absence of information and rumours cause anxiety and fear [26]. Common psychological issues during post-lockdown period were anxiety, insomnia, and behavioural issues which were suggestive of late psychological manifestations such as post-traumatic stress disorder and other trauma-related psychological sequelae. Zandifar and Badrfam highlighted the role of unpredictability, uncertainty, seriousness, and social isolation in contributing to stress and mental morbidity [27].

The prominent social problems of “high expressed emotions” (17%) by the patient’s relatives during hospitalization could be due to the novel nature of COVID-19 infection, uncertain and frequently changing clinical picture, and diagnostic and treatment guidelines. Grover et al. also found family members (38.8%) have anxiety [8]. Shigemura et al. pronounced families as “vulnerable populations” needing particular efforts [26].

Social problems related to containment zones like non-access to daily necessities were reported by 13.6% of hospitalized patients and 30% of discharged patients. With the progression of the pandemic and the spread of infection, more patients were diagnosed with COVID-19 resulting in the formation of more containment zones. Often, sequential infection of multiple family members one after another led to non-removal of the containment zone, hence prolonging its duration, consequently 25.4% of discharged patients reported problems of containment lasting longer than expected and 18% of discharged patients reported social/physical restriction, quarantined at home resulting in a feeling of boredom and loneliness. It was an interesting finding that boredom and loneliness were equally reported by both youth and the elderly. Grover et al. also reported feelings of loneliness in 21.3% of participants [8]. Xiang et al. have opined that quarantine can induce loneliness, boredom, anger, anxiety, and depression [28]. In addition, quarantined people lose face-to-face connections and traditional social interventions, and this is a stressful phenomenon [29].

As predicted, the government’s efforts for financial support and food security will be insufficient [23]; in our study, social problems due to financial matters were reported by 17.4% of discharged patients as opposed to very less patients during hospitalization. It is understandable as most of the patients belonged to the middle or low socioeconomic class, and didn't have enough savings to last more than a few days. According to Blustein, people with precarious work experience uncertainty and chronic stress, putting them at risk for mental and physical health [30]. These risk factors may further worsen the COVID-19 crisis while simultaneously exposing inequities that existed before the crisis.

Social problems of discrimination and stigma were reported by 6.4% of hospitalized patients and 5.8% of discharged patients. It can be proposed that once patients were labelled as COVID-19 positive, they remained so even after discharge or being COVID-19 negative on repeat testing. Grover et al. also reported that 10% of participants face stigma [8].

An isolated and peculiar social problem of a longer wait for the report of repeat COVID-19 test before discharge was reported by 11% of hospitalized patients. It was noted during the early pandemic when repeat COVID-19 testing was advised before discharge. Since our institutional laboratory was recently established and was also catering to the needs of adjoining districts hence overloaded which led to this issue. Accordingly, 6.2% of patients reported late results of family members or contacts. In the same line, 5.6% of discharged patients requested for medical certification for various purposes such as to appear in exams, fitness for jobs, or travelling. As with all over the world, our medical and non-medical staff was also already working ways beyond their capacity and were overburdened, therefore such non-emergency requests could not be addressed immediately. Many studies have corroborated this finding. Khasne et al. reported that 52.8% of healthcare workers had pandemic-related burnout [16]. The doctors were 1.64 times and the support staff were five times more prone to burnout. A Lancet editorial raised this issue “India’s response has been constrained by a shortage of health workers, but this should be remedied by new reforms that would mobilize additional healthcare workers from different sources” [23].

During hospitalization 10% of patients, mainly females, were distressed because nobody else was there at home to take care of household chores. This decreased to 2.4% after discharge. This can be explained on the basis of prevalent gender-assigned roles and possible gender discrimination in India. A similar socio-economic factor of poverty was mirrored in 4.4% of discharged patients who reported difficulty isolation due to less number of rooms at home. This is important as implementing public health measures is difficult in places with overcrowded living conditions and inadequate hygiene and sanitation [23].

During hospitalization, 7% of patients misbehaved with medical staff. Some of them were spreading rumours and making fake videos about the hospital administration, and being abusive and physically aggressive. Similarly, 3.4% of patients didn't follow COVID-19-related protocols. All of these can be explained as a result of lack of trust in the healthcare system, misinformation, rumours, lack of education and irresponsible media reporting. Panigrahi et al. in their online survey found that 64.8% of participants felt the steps of the government were inadequate for the prevention and control of COVID-19 [11].

We recommend from our learning from this study that while preparing and managing the pandemic, planners and policymakers must keep a holistic approach. The psychological and social dimensions are important determinants of health; therefore while planning confinement strategies during pandemics or outbreaks, various social and psychological issues must be thought about and addressed timely. Psychological and social health-related information must be considered in guidelines, to minimize myths and misconceptions. The day-to-day difficulties faced by citizens, patients, or family members should be addressed by setting up command and monitoring cells.

The main strength and distinguishing feature of our study was that we studied the psychological and social problems encountered by the patients infected with COVID-19 during hospitalization and after discharge from the hospital. These patients were directly interviewed by the psychiatrists daily during the hospitalization and post-discharge period. No such studies have been done in India. However, there are certain limitations. This was a retrospective study and the data was pre-recorded; hence, a few other important information may have been missed. Being a single-centre, hospital-based study, the findings may not be generalizable.

## Conclusions

The present study observed the significant burden of psychological and social issues in hospitalized COVID-19 patients even after their discharge from hospital. Often psychological and social health is overlooked, which may be more so during pandemics as found in our study. These overlooked issues may pre-dispose, precipitate, complicate, or even present as physical symptoms or medical illnesses. If not identified and addressed adequately, these may hinder the management and pose an additional burden on individual, community, and administration levels. It may also act as an unidentified healthcare burden, further weakening the healthcare system, especially in countries like India. Additionally, various unrehearsed mitigation strategies, such as lockdown, quarantine, social isolation, containment zone, etc., to curtail pandemics may unintentionally lead to various psychological and social problems. Future pandemic preparedness should seek lessons from the current COVID-19 pandemic.
